# Effects of the Highly COX-2-Selective Analgesic NSAID Etoricoxib on Human Periodontal Ligament Fibroblasts during Compressive Orthodontic Mechanical Strain

**DOI:** 10.1155/2019/2514956

**Published:** 2019-03-10

**Authors:** Christian Kirschneck, Erika Calvano Küchler, Michael Wolf, Gerrit Spanier, Peter Proff, Agnes Schröder

**Affiliations:** ^1^Department of Orthodontics, University Hospital Regensburg, Franz-Josef-Strauss-Allee 11, 93053 Regensburg, Germany; ^2^Professor of the Post-Graduation Program in Pediatric Dentistry, School of Dentistry of Ribeirão Preto, University of São Paulo, Avenida do Café, s/n Campus da USP, Ribeirão Preto/SP CEP: 14040-904, Brazil; ^3^Department of Orthodontics, University Hospital RWTH Aachen, Pauwelsstraße 30, 52074 Aachen, Germany; ^4^Department of Cranio-Maxillo-Facial Surgery, University Hospital Regensburg, Franz-Josef-Strauss-Allee 11, 93053 Regensburg, Germany

## Abstract

Human periodontal ligament (hPDL) fibroblasts play a major role during periodontitis and orthodontic tooth movement, mediating periodontal inflammation, osteoclastogenesis, and collagen synthesis. The highly COX-2-selective NSAID etoricoxib has a favorable systemic side effect profile and high analgesic efficacy, particularly for orthodontic pain. In this in vitro study, we investigated possible side effects of two clinically relevant etoricoxib concentrations on the expression pattern of mechanically strained hPDL fibroblasts and associated osteoclastogenesis in a model of simulated orthodontic compressive strain occurring during orthodontic tooth movement. hPDL fibroblasts were incubated for 72 h under physiological conditions with etoricoxib at 0 *μ*M, 3.29 *μ*M, and 5.49 *μ*M, corresponding to clinically normal and subtoxic dosages, with and without mechanical strain by compression (2 g/cm^2^) for the final 48 h, simulating conditions during orthodontic tooth movement in compressive areas of the periodontal ligament. We then determined gene and/or protein expression of COX-2, IL-6, PG-E_2_, RANK-L, OPG, ALPL, VEGF-A, P4HA1, COL1A2, and FN1 via RT-qPCR, ELISA, and Western blot analyses as well as apoptosis, necrosis, cell viability, and cytotoxicity via FACS, MTT, and LDH assays. In addition, hPDL fibroblast-mediated osteoclastogenesis was assessed by TRAP staining in coculture with RAW267.4 cells for another 72 h. Gene and protein expression of all evaluated factors was significantly induced by the mechanical compressive strain applied. Etoricoxib at 3.29 *μ*M and 5.49 *μ*M significantly inhibited PG-E_2_ synthesis, but not COX-2 and IL-6 gene expression nor RANK-L-/OPG-mediated osteoclastogenesis or angiogenesis (VEGF-A). Extracellular matrix remodeling (COL1A2, FN1) and bone anabolism (ALPL), by contrast, were significantly stimulated particularly at 5.49 *μ*M. In general, no adverse etoricoxib effects on hPDL fibroblasts regarding apoptosis, necrosis, cell viability, or cytotoxicity were detected. Clinically dosed etoricoxib, that is, a highly selective COX-2 inhibition, did not have substantial effects on hPDL fibroblast-mediated periodontal inflammation, extracellular matrix remodeling, RANK-L/OPG expression, and osteoclastogenesis during simulated orthodontic compressive strain.

## 1. Introduction

The human periodontal ligament (hPDL) fibroblast is the major cell type occurring within the periodontal ligament, a fiber- and cell-rich connective tissue, which adjoins teeth to the surrounding alveolar bone of the jaws [1, 2]. The resulting dentoalveolar joint of limited mobility (gomphosis) enables a physiological transformation of otherwise bone-catabolic masticatory compressive forces into tensile anabolic forces via specifically aligned collagen fibers connecting the tooth cementum to the cortical inner bony surface of the tooth socket [2, 3]. Due to its high perfusion rate, the periodontal ligament also sustains the surrounding tissue and numerous embedded nerve endings and receptors are involved in proprioception and the regulation of mastication [2].

hPDL fibroblasts have been shown to play an important role not only in homeostasis and turnover of the periodontal ligament [4] but also in the mediation and regulation of various physiological and pathological processes [1, 5, 6]. In particular, they are known to react to bacterial toxins and lipopolysaccharides of periodontal pathogens, which are recognized by Toll-like receptors, with increased expression and release of inflammatory cytokines, chemokines, and other factors leading to increased osteoclastogenesis via the also upregulated RANK-L/OPG system [7] and causing uncontrolled loss of periodontal attachment and bone (periodontitis) [8, 9]. Apart from toxins, also mechanical stimuli by compressive or tensile forces have been shown to trigger similar reactions [6, 10], possibly mediated by integrin-associated mechanotransduction via focal adhesions [11].

In the dental discipline of orthodontics, these hPDL fibroblast-mediated pseudoinflammatory, multicellular, immunological processes are necessary to enable controlled osteoclastogenesis to move teeth through the alveolar bone [6, 11]. Orthodontic tooth movement is induced therapeutically by applying mechanical forces to the teeth to correct pathologies in tooth position [6, 12], being a contributing factor for various oral diseases such as caries [13] and periodontitis [14]. In addition, remodeling processes of the extracellular matrix as well as increased angiogenesis, osteoblastogenesis, and bone formation, mediated by hPDL fibroblasts [1, 4, 6], play an integral part in orthodontic tooth movement [11].

Various pharmacologically active substances have been shown to influence the expression pattern of hPDL fibroblasts and hPDL fibroblast-mediated osteoclastogenesis during pathological periodontal inflammation (periodontitis) [15] as well as physiological orthodontic tooth movement [10, 16–18], that is, mechanical compressive and tensile strain. Drugs can thus have a significant attenuating or propagating effect on the regulation of these processes, which can be either desirable or detrimental for treatment purposes [19]. Nonsteroidal anti-inflammatory drugs (NSAID) are among the most frequently used analgesics worldwide, often available without prescription over the counter and used for the treatment of pain, fever, and inflammation in general as well as chronic inflammatory diseases such as rheumatoid arthritis [19, 20]. In orthodontics, these are also used to treat orthodontically induced pain sensations, which can otherwise prompt termination or rejection of treatment [21]. Furthermore, due to an increasing number of adult patients opting for orthodontic treatment [22], regular systemic medication with NSAID for other medical conditions is encountered more and more frequently in orthodontic practice [19].

Etoricoxib, which has been approved in Europe for clinical use in the treatment of various inflammatory diseases as well as postoperative dental pain, belongs to a special subgroup of NSAID called coxibs, which particularly target the inflammation-induced cyclooxygenase isoform 2 (COX-2) over the constitutively expressed isoform 1 (COX-1) to various degrees [23] with etoricoxib being the most COX-2-selective coxib currently available (COX-2/1 ratio of 344 : 1) one [24, 25]. Apart from being the only coxib approved for use in dental context, it has been shown to have superior analgesic efficacy with a favorable side effect profile compared to other NSAID [26, 27], particularly regarding orthodontic pain [28, 29]. For this reason, it has been identified as potential analgesic of choice in orthodontics [29]. Since prostaglandin release by hPDL fibroblasts has been shown to play an important role during the pseudoinflammatory molecular processes enabling orthodontic tooth movement [30–32], an etoricoxib-induced inhibition of prostaglandin synthesis might, however, have deleterious effects on hPDL fibroblast-mediated osteoclastogenesis and thus tooth movement, possibly limiting its suitability as analgesic in orthodontics.

At present, no studies are available investigating the effects of etoricoxib on hPDL fibroblast-mediated periodontal inflammation, osteoclastogenesis, angiogenesis, and turnover of the extracellular matrix and bone formation occurring during orthodontic tooth movement induced by mechanical strain on hPDL fibroblasts. Previous in vitro and animal studies on other NSAIDs indicate suppressive effects [33] with available data; however, they are conflicting [15, 19, 20, 23]. This is most likely due to dose dependencies of pharmacodynamic effects or a possibly different relative significance of COX-2 and COX-1 in the mediation of the pseudoinflammatory processes in the orthodontic context, which merits further investigation.

The aim of this study was therefore to elucidate the effects of etoricoxib on the hPDL fibroblast expression pattern and hPDL fibroblast-mediated osteoclastogenesis in the presence and absence of physiological mechanical compressive strain, occurring during orthodontic tooth movement, at cell medium concentrations corresponding to clinically administered dosages and associated local tissue concentrations.

## 2. Material and Methods

### 2.1. hPDL Fibroblast Cultivation and Origin

Pooled primary human periodontal ligament (hPDL) fibroblast cell lines from six patients (3 females, 3 males; age: 17-27 years) were extracted from periodontal connective tissue of human caries-free wisdom teeth, which were surgically removed for medical reasons in our facility. The cultivation and experimental usage of these human cells were approved by the ethics committee of the University of Regensburg, Germany (approval number 12-170-0150). To establish hPDL fibroblast cell lines, we scraped connective tissue residues off the middle third of the roots and transferred them to 6-well plates, cultivated under standard cell culture conditions (37°C, 5% CO_2_, and 100% H_2_O) in full medium (DMEM high glucose, D5796, Sigma-Aldrich, Munich, Germany), supplemented with 10% FCS (P30-3306, PAN-Biotech, Aidenbach, Germany), 1% L-glutamine (SH30034.01, GE Healthcare Europe, Munich, Germany), 100 *μ*M ascorbic acid (A8960, Sigma-Aldrich), and 1% antibiotics/antimycotics (A5955, Sigma-Aldrich). We confirmed the hPDL fibroblast lineage according to the expression of PDL-specific marker genes (PCR amplification and agarose gel electrophoresis) and spindle-shaped morphology [34] (Supplementary Figure/Table 1). Until use, all hPDL fibroblasts were stored in liquid nitrogen (90% FCS, 10% DMSO, and freezing at 1°C/minute).

### 2.2. Experimental Design

**Figure 1:**
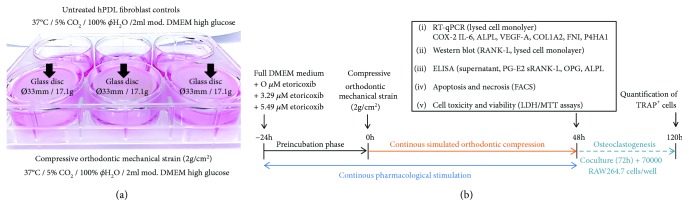
(a) *In vitro* model for the simulation of compressive orthodontic mechanical strain. hPDL: human periodontal ligament. (b) Timeline of the *in vitro* experiments performed and ponding outcomes.

At a density of 70000 cells in 2 ml DMEM per well, pooled hPDL fibroblasts (3-5th passage) were seeded onto 6-well cell culture plates. For RT-qPCR analyses as well as LDH/MTT assays, hPDL fibroblasts in six experimental groups with 9 wells (*n* = 9) on three plates (*N* = 3) each were, respectively, incubated with either 0 *μ*M (control), 3.29 *μ*M, or 5.49 *μ*M etoricoxib (Etoricoxib VETRANAL™ analytical standard: 32097 FLUKA, Sigma-Aldrich®, Taufkirchen, Germany) for 72 h, corresponding to assumed local concentrations reached in the PDL during normal and subtoxic clinical dosing in man [35, 36], with or without (3/3 wells per plate) compressive mechanical strain of 2 g/cm^2^ for 48 h after a 24 h preincubation phase by means of a glass disc according to a published and well-established protocol for the simulation of compressive orthodontic mechanical strain [30, 34] (Figure 1(a)). RANK-L Western blot was performed for seven (*N* = 7), ELISA for six (*N* = 2, *n* = 6), and FACS analyses in duplicates for three (*N* = 3, *n* = 6) biological replicates (Figure 1(b)).

### 2.3. Real-Time Polymerase Chain Reaction (RT-qPCR) for Relative Gene Expression Quantification

RNA isolation and quality assessment as well as RT-qPCR were performed as described before according to MIQE guidelines [34]. Total RNA was retrieved from hPDL fibroblasts by administering 1 ml peqGOLD TriFast™ (PEQLAB Biotechnology GmbH, Erlangen, Germany) per well and further handling according to the manufacturer's instructions. The obtained RNA pellet was eluted in 25 *μ*l nuclease-free water (T143, BioScience Grade, Carl Roth GmbH & Co. KG, Karlsruhe, Germany) and immediately cooled on ice. A standardized quantity of 1 *μ*g RNA per sample was transcribed in cDNA as described before [34]. A Mastercycler® ep realplex-S thermocycler (Eppendorf AG, Hamburg, Germany) was used for RT-qPCR as described before [34]. Each reaction mix comprised 7.5 *μ*l SYBR® Green JumpStart™ Taq ReadyMix™ (S4438; Sigma-Aldrich, Munich, Germany) as well as 1.5 *μ*l of the respective cDNA solution (dilution 1 : 10) and 7.5 pmol (0.75 *μ*l) of the respective primer pair (3.75 pmol/primer). To achieve a total amount of 15 *μ*l, nuclease-free H_2_O (T143, Carl Roth GmbH & Co. KG) was added accordingly with all components (except cDNA) prepared as a master mix to minimize manual pipetting-related technical errors. cDNA amplification was performed in 45 cycles (95°C/5 min initial heat activation, 95°C/10 s per cycle denaturation, 60°C/8 s annealing, and 72°C/8 s extension). At the end of each extension step, SYBR Green I fluorescence was quantified at 521 nm and *C*_*q*_ values were identified as second derivative maximum of the fluorescence signal curve employing the software realplex (version 2.2, Eppendorf AG, CalQPlex algorithm, Automatic Baseline, Drift Correction On). Normalization of target genes for assessment of relative gene expression was based on two reference genes (RPL22/PPIB), which were validated before for hPDL fibroblasts and the *in vitro* model used [34]. We calculated relative gene expression as 2^−Δ*Cq*^ [37] with *∆C*_*q*_ = *C*_*q*_ (target gene)–*C*_*q*_ (mean RPL22/PPIB) according to MIQE guidelines [34]. RT-qPCR primer design and validation were performed according to MIQE quality criteria as described before [34] (Table 1, Supplementary Table 2).

### 2.4. Western Blot Quantification of RANK-L Protein Expression

Total protein was extracted from hPDL fibroblasts on ice by applying 100 *μ*l CelLytic M (C2978; Sigma-Aldrich) per well with additional proteinase inhibitors (Carl Roth GmbH & Co. KG) and quantified by Roti-Quant (K015.3; Carl Roth GmbH & Co. KG) as recommended by the manufacturer. Equal protein amounts were then separated by reducing SDS polyacrylamide gel electrophoresis (12%) and electro blotted onto a polyvinylidene difluoride (PVDF) membrane, blocked with 5% nonfat milk in tris-buffered saline and 0.1% Tween 20, pH 7.5 (TBS-T) at 4°C overnight. For 1 h, anti-RANK-L (1 : 2000, ABIN500805, antibodies-online, Aachen, Germany) or anti-HSP90 (1 : 500, Santa Cruz Biotech, Heidelberg, Germany) was incubated in 0.5% milk in TBS-T. After 3x TBS-T washing and blots were further incubated for 1 h at room temperature with horseradish peroxidase-conjugated anti-rabbit IgG (1 : 5000, Pierce, Rockford, USA) in 0.5% milk in TBS-T. Antibody binding was visualized by an enhanced chemiluminescence system (Pierce, Rockford, USA), and bands were quantified densitometrically with ImageJ (ver. 1.47, Wayne Rasband, National Institutes of Health, USA).

### 2.5. Enzyme-Linked Immunosorbent Assay (ELISA) of PG-E_2_, sRANK-L, OPG, and ALPL

To quantify the concentration of prostaglandin E_2_ (PG-E_2_), soluble receptor activator of nuclear factor kappa b ligand (sRANK-L), osteoprotegerin (OPG), and alkaline phosphatase (ALPL) in the cell culture supernatant per well, which was related to the respective number of hPDL fibroblasts determined with a Beckman Coulter counter (Z2 cell counter), ELISAs were performed as recommended by the manufacturers' instructions (PGE_2_: 514010, Cayman Chemical, Ann Arbor, USA; RANK-L: RD193004200R, BioVendor, Brno, Czech Republic; OPG: EHTNFRSF11B, Thermo Fisher Scientific; ALPL: OKEH00757, Aviva Systems Biology, San Diego, USA).

### 2.6. hPDL-Mediated Osteoclastogenesis in Coculture Assessed by TRAP Histochemistry

Stimulated hPDL fibroblasts were washed with PBS at the end of the 72 h incubation phase. We then added a macrophage cell line (immortal RAW264.7 cells, CLS Cell Lines Service, Eppelheim, Germany) at a concentration of 70000 cells per well. By adding the RAW cells after the phase of mechanical strain, we avoided a possibly biasing force induction of RAW cell differentiation [10]. We incubated the resulting coculture for another 72 h under cell culture conditions [10, 16], enabling osteoclastogenesis induced by hPDL fibroblasts [38]. Differentiated mononucleated and multinucleated TRAP-positive cells were considered to be osteoclast precursor and osteoclast-like cells, respectively [38, 39], as identified by histochemical TRAP staining (tartrate-resistant acid phosphatase, red) with TRAP-positive cells counted by a blinded investigator at ×100 (Olympus IX50 microscope, Olympus, Germany) in ten random fields of view per well (biological replicate). The arithmetic mean was employed for further analysis [10].

### 2.7. Cell Apoptosis and Necrosis as Assessed by Flow Cytometry

FITC (fluorescein isothiocyanate) annexin V (apoptosis marker) and propidium iodide (PI, necrosis marker) stainings were carried out according to the manufacturer's instructions (FITC Annexin V Apoptosis Detection Kit I, 556547, BD Pharmingen, Heidelberg, Germany), followed by FACS (fluorescence-activated cell sorting, BD FACSCalibur, Heidelberg, Germany). Membrane-adherent hPDL fibroblasts were washed twice with PBS (phosphate-buffered saline) of 4°C and immediately resuspended at 10^6^ cells/pro ml (Z2 cell counter, Beckman Coulter, Krefeld, Germany) in 10x binding buffer (0.1 M HEPES/NaOH pH7.4, 1.4 M NaCl, 25 mM CaCl_2_). 100 *μ*l was then transferred to a 5 ml FACS tube, and 5 *μ*l FITC annexin V and 5 *μ*l PI were added. hPDL fibroblasts were gently vortexed and incubated in the dark at room temperature for 15 min. Prior to flow cytometry, 400 *μ*l binding buffer was added to each tube (FSC: 5 V, SSC: 349 V, FITC: 350 V, PerCP-Cy5-5: and 450 V; Threshold FSC: 5000; Laser Delay Blue: 0.00, Red: 24.10; Area scaling Blue: 1.79, Red: 1.80; FSC Area Scaling: 1.08).

### 2.8. Cell Cytotoxicity as Assessed by LDH Assay

Commercially available lactate dehydrogenase (LDH) assays (04744926001, Roche, Mannheim, Germany) were used according to the manufacturer's instructions. 100 *μ*l freshly prepared LDH solution (22 *μ*l catalyst mixed with 1 ml dye) was added to 100 *μ*l supernatant and incubated in the dark at room temperature for 30 min before adding 50 *μ*l of stop solution. An ELISA reader (Multiskan GO Microplate Spectrophotometer, Thermo Fisher Scientific), was used to measure LDH activity (absorbance at 490 nm), subtracting background absorbance at 690 nm.

### 2.9. Cell Viability (Mitochondrial Enzymatic Activity) as Assessed by MTT Assay

For MTT (3-(4,5-dimethylthiazol-2-yl)-2,5-diphenyltetrazoliumbromid) assays, 400 *μ*l MTT solution in PBS (5 mg/ml, 4022.1, Carl Roth GmbH & Co. KG) was added per well for the final five hours of incubation. After, removal of the 1 ml DMSO per well was added and incubation continued at 37°C for another 5 min measuring final absorbance (cell viability) at 550 nm with an ELISA reader (Multiskan GO Microplate Spectrophotometer, Thermo Fisher Scientific).

### 2.10. Statistical Analysis

Prior to statistical analysis, all absolute data values were divided by the respective arithmetic mean of the pressure-untreated 0 *μ*M etoricoxib controls to obtain normalized data values relative to these controls, set to 1. Using the software application SPSS® Statistics 24 (IBM®, Armonk, NY, USA), all data were tested for normal distribution (Shapiro-Wilk test) and homogeneity of variance (Levene's test). Descriptive statistics are given as mean ± standard deviation (*M* ± SD). The experimental groups were compared by one-way ANOVAs with Tukey HSD post hoc tests for pairwise comparisons. In case of variance heterogeneity, an adjustment by Welch's test and Games-Howell post hoc tests was used. All differences were considered statistically significant at *p* ≤ 0.05.

Sufficient statistical power was evaluated by an a-priori power analysis with G^∗^Power (version 3.1.9.2) [40], based on a clinically relevant 20% etoricoxib-induced reduction of the mean- (±SD-) expected mechanical strain-elevated PG-E_2_ concentration in the cell culture supernatant of 172.9 pg (±17.5 pg) per 100.000 cells (*d* = 1.976) [41] for independent ANOVA post hoc *t*-test and Mann-Whitney *U* tests, achieving an actual power of 86.9% and 84.9% at *α* = 5% and *β* = 20% for the minimally used sample size of *n* = 6 per experimental group.

## 3. Results

### 3.1. Effects of Etoricoxib and Compressive Orthodontic Mechanical Strain on the Expression and Secretion of Proinflammatory Factors

**Figure 2:**
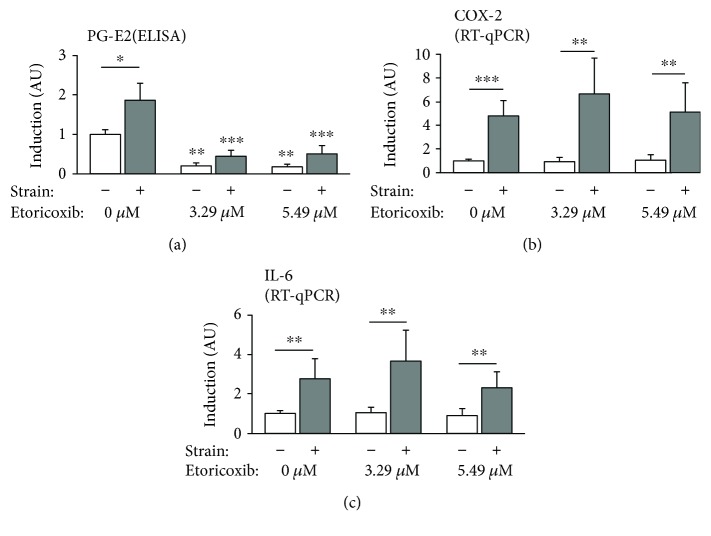
Relative secretion of prostaglandin E_2_ (a) as well as expression of proinflammatory genes cyclooxygenase 2 (b) and interleukin 6 (c) as normalized x-fold induction relative to 0 *μ*M etoricoxib without mechanical strain. Asterisms without bars indicate significant differences to the corresponding 0 *μ*M control with or without strain. ^∗^ *p* ≤ 0.05, ^∗∗^ *p* ≤ 0.01, and ^∗∗∗^ *p* ≤ 0.001. AU: arbitrary units. Bars show mean and standard deviation. PG-E_2_ ELISA: *N* = 2, *n* = 6; RT-qPCR: *N* = 3, *n* = 9.

Prostaglandin E_2_ (PG-E_2_) secretion (ELISA, Figure 2(a)) was significantly regulated both by the mechanical strain applied to the hPDL cells and by etoricoxib (*F* = 55.08; df_1/2_ = 5/13.56, *p* < 0.001). Force application significantly increased PG-E_2_ synthesis in the control group (*p* = 0.024), whereas etoricoxib at cell medium concentrations of 3.29 *μ*M and 5.49 *μ*M significantly inhibited PG-E_2_ synthesis in both the presence (*p* = 0.002) and absence (*p* < 0.001) of mechanical compressive strain compared to the respective 0 *μ*M controls.

Relative gene expression of cyclooxygenase 2 (COX-2) (Figure 2(b), *F* = 22.366, df_1/2_ = 5/20.4, *p* < 0.001) and interleukin 6 (IL-6, Figure 2(c), *F* = 13.399, df_1/2_ = 5/21.024, *p* < 0.001) was significantly induced by compressive force application at all etoricoxib concentrations tested (COX-2: *p* ≤ 0.009, IL-6: *p* ≤ 0.01), which did have no significant influence on the level of COX-2 (*p* ≥ 0.502) and IL-6 (*p* ≥ 0.712) gene expression in neither the presence or absence of compressive force.

### 3.2. Effects of Etoricoxib and Compressive Orthodontic Mechanical Strain on the RANK-L/OPG System and hPDL-Mediated Osteoclastogenesis

**Figure 3:**
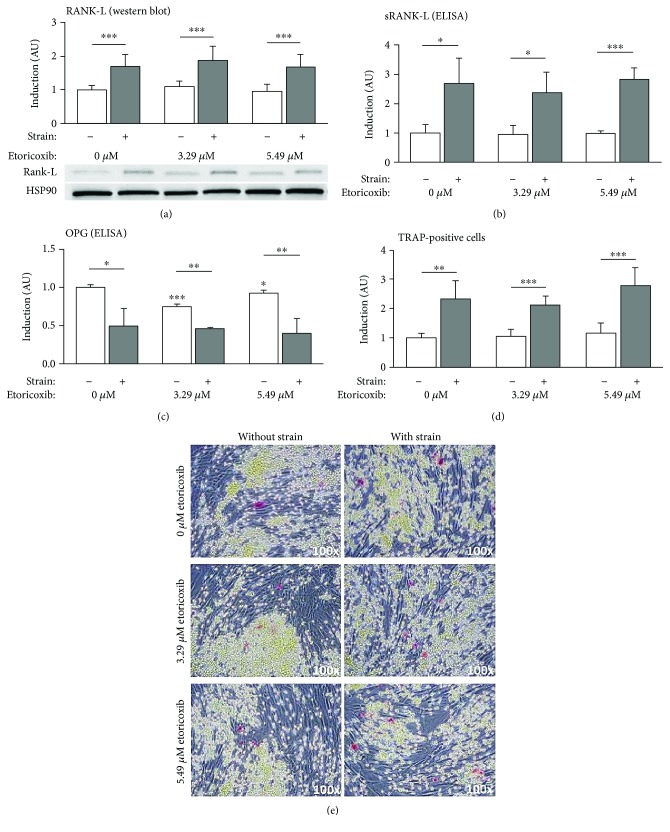
Relative expression of RANK-L/OPG and extent of hPDL fibroblast-mediated osteoclastogenesis. (a) RANK-L expression according to Western blot densitometry (*N* = 7), (b) sRANK-L and (c) OPG protein secretion according to ELISA (*N* = 2, *n* = 6), and (d, e) osteoclastogenesis after coculture with RAW264.7 cells (TRAP staining and mono- and multinucleated osteoclast precursor and osteoclast-like TRAP-positive cells in red, *N* = 2, *n* = 6), given as normalized x-fold induction relative to 0 *μ*M etoricoxib without mechanical strain. Asterisms without bars indicate significant differences to the corresponding 0 *μ*M control. Abbreviations: see manuscript text. ^∗^ *p* ≤ 0.05, ^∗∗^ *p* ≤ 0.01, and ^∗∗∗^ *p* ≤ 0.001. AU: arbitrary units. Bars show mean and standard deviation; TRAP: tartrate-resistant acid phosphatase.

Expression of the osteoclastogenesis-stimulating factor RANK-L (receptor activator of nuclear factor kappa b ligand) at the protein level was significantly induced by the mechanical strain applied at all etoricoxib concentrations evaluated (*p* ≤ 0.029), both in Western blot analysis (Figure 3(a), *F* = 13.21, df = 5, *p* < 0.001) and in ELISA (Figure 3(b), *F* = 27.614, df_1/2_ = 5/12.490, *p* < 0.001). Etoricoxib, on the other hand, had no significant impact on RANK-L production in the presence (*p* ≥ 0.907) or absence (*p* ≥ 0.985) of compressive force, as confirmed by both analyses. Expression of the RANK-L antagonist osteoprotegerin (OPG) (Figure 3(c), *F* = 72.461, df_1/2_ = 5/13.503, *p* < 0.001) at the protein level was significantly suppressed by the compressive force applied at all etoricoxib concentrations (*p* ≤ 0.011), which had a significant, but only minimal, inhibitory effect in the absence of mechanical strain (*p* ≤ 0.028), which was, however, not detectable during compression (*p* ≥ 0.863). hPDL-mediated osteoclastogenesis in coculture (Figure 3(d) and 3(e), *F* = 33.758, df_1/2_ = 5/21.573, *p* < 0.001), as determined by TRAP staining, was significantly induced by the simulated orthodontic force application independent of etoricoxib (*p* ≤ 0.002), which had no significant influence on osteoclastogenesis, at a concentration of neither 3.29 *μ*M (*p* ≥ 0.947) nor 5.49 *μ*M (*p* ≥ 0.638).

### 3.3. Effects of Etoricoxib and Compressive Orthodontic Mechanical Strain on Bone Formation, Angiogenesis, and Extracellular Matrix Remodeling

**Figure 4:**
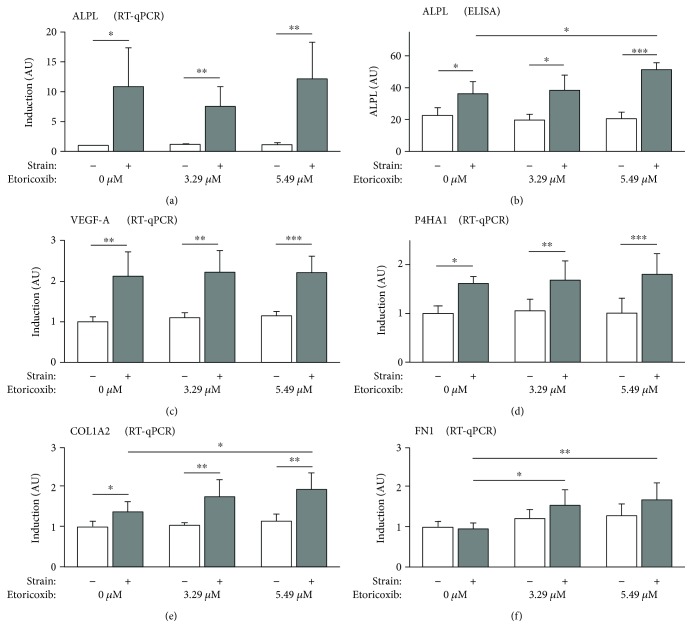
Relative expression of alkaline phosphatase (a, b) (bone formation), as well as of VEGF-A (c) (angiogenesis) and P4HA1, COL1A2, and FN1 (d), (e), and (f), respectively (extracellular matrix remodeling), except for ELISA as normalized x-fold induction relative to 0 *μ*M etoricoxib without mechanical strain. Abbreviations: see manuscript text. ^∗^ *p* ≤ 0.05, ^∗∗^ *p* ≤ 0.01, and ^∗∗∗^ *p* ≤ 0.001. AU: arbitrary units. Bars show mean and standard deviation. ELISA: *N* = 2, *n* = 6; RT-qPCR: *N* = 3, *n* = 9; ELISA: enzyme-linked immunosorbent assay; RT-qPCR: real-time reverse transcription quantitative polymerase chain reaction.

Relative gene expression of alkaline phosphatase (ALPL) (Figure 4(a), *F* = 14.555, df_1/2_ = 5/20.155, *p* < 0.001), which is known to be a biochemical marker of bone formation and mineralization [39], was significantly elevated during the application of compressive forces at all etoricoxib concentrations (*p* ≤ 0.016), which was confirmed at the protein level by ELISA (*p* ≤ 0.046, Figure 4(b), *F* = 41.63, df_1/2_ = 5/13.826, *p* < 0.001). 5.49 *μ*M etoricoxib at concurrent force application induced a significant increase in ALPL expression at the translational level (*p* = 0.029), which was not detectable at the mRNA level (*p* = 0.998), with no other significant etoricoxib effects observed (*p* ≥ 0.568).

hPDL fibroblast-mediated angiogenesis, as determined by relative gene expression of vascular endothelial growth factor A (VEGF-A) (Figure 4(c), *F* = 24.976, df_1/2_ = 5/21.674, *p* < 0.001), showed a significant force-associated induction independent of etoricoxib (*p* ≤ 0.004), which did have no significant effects at neither of the two concentrations applied 3.29 *μ*M (*p* ≥ 0.5) and 5.49 *μ*M (*p* ≥ 0.117).

Collagen synthesis and extracellular matrix remodeling, represented by relative gene expression of prolyl 4-hydroxylase subunit alpha-1 (P4HA1) (Figure 4(d), *F* = 35.732, df_1/2_ = 5/21.685, *p* < 0.001), alpha-2 subunit of the fibril-forming type I collagen (COL1A2) (Figure 4(e), *F* = 14.354, df_1/2_ = 5/20.725, *p* < 0.001), and fibronectin 1 (FN1) (Figure 4(f), *F* = 8.676, df_1/2_ = 5/21.859, *p* < 0.001), were significantly stimulated by compressive force application at all etoricoxib concentrations tested, except for FN1 (*p* ≥ 0.236, P4HA1 *p* ≤ 0.023, and COL1A2 *p* ≤ 0.016). During compression, 3.29 *μ*M etoricoxib significantly elevated FN1 (*p* = 0.016) and 5.49 *μ*M etoricoxib FN1 (*p* = 0.005) and COL1A2 (*p* = 0.042) gene expression, whereas P4HA1 gene expression was not significantly influenced by etoricoxib at the concentrations tested 3.29 *μ*M (*p* ≥ 0.464) and 5.49 *μ*M (*p* ≥ 0.839).

### 3.4. Apoptosis, Necrosis, Cell Viability, and Etoricoxib Cytotoxicity

**Figure 5:**
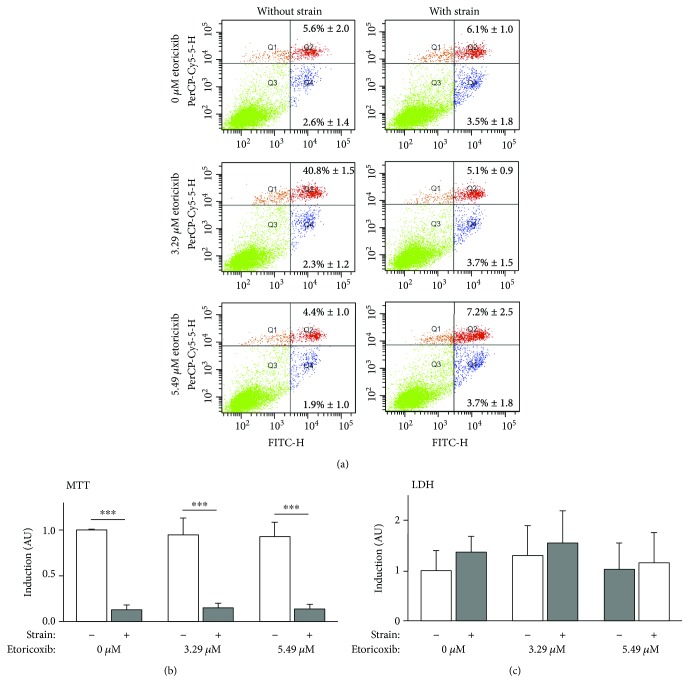
Apoptosis and necrosis (FACS), cell viability (MTT), and cytotoxicity (LDH) of hPDL fibroblasts incubated with rising etoricoxib concentrations with and without mechanical strain. (a) Representative FACS distributions (*M* ± SD, positive cells relative to total cells (%)) of apoptotic (Q4, FITC annexin V), necrotic (Q2, propidium iodide), and viable cells (Q3). *N* = 3, *n* = 6. (b) Cell viability as assessed by MTT assay; *N* = 3, *n* = 9. (c) Cytotoxicity assessed by LDH assay; *N* = 3, *n* = 9. All values given as normalized x-fold induction relative to 0 *μ*M etoricoxib without mechanical strain. ^∗^ *p* ≤ 0.05, ^∗∗^ *p* ≤ 0.01, and ^∗∗∗^ *p* ≤ 0.001. AU: arbitrary units. Bars show mean and standard deviation. PerCP-Cy5-5-H: peridinin-chlorophyll-protein complex channel (propidium iodide, necrosis); FITC-H: fluorescein thiocyanate channel (annexin V, apoptosis); MTT: 3-(4,5-dimethylthiazol-2-yl)-2,5-diphenyltetrazoliumbromid; LDH: lactate dehydrogenase.

As determined by FACS analysis (Figure 5(a)), the percentage of apoptotic (Q4, *F* = 1.664, df = 5, *p* = 0.174) and necrotic (Q2, *F* = 2.524, df = 5, *p* = 0.051) cells was not significantly increased by compressive force application or etoricoxib independent of the concentration tested. As an exception, 5.49 *μ*M etoricoxib and concurrent mechanical strain evoked a significant slight increase in the number of necrotic cells (7.2%, *p* = 0.041) compared to 5.49 *μ*M without pressure application (4.4%).

Cell viability, that is, mitochondrial enzymatic activity, as assessed by MTT assay (Figure 5(b), *F* = 904.714, df_1/2_ = 5/20.707, *p* < 0.001), was significantly reduced during compression at all etoricoxib concentrations tested (*p* < 0.001), whereas etoricoxib did not affect cell viability neither at 3.29 *μ*M (*p* ≥ 0.945) nor at 5.49 *μ*M (*p* ≥ 0.74).

No significant cytotoxicity on hPDL fibroblasts, as assessed by LDH assay (Figure 5(c), *F* = 1.495, df = 5, *p* = 0.209), was detected for either pressure application (*p* ≥ 0.662) or etoricoxib at 3.29 *μ*M (*p* ≥ 0.821) or 5.49 *μ*M (*p* ≥ 0.607).

## 4. Discussion

In our in vitro study on human periodontal ligament (hPDL) fibroblasts, we investigated possible effects of etoricoxib at cell medium concentrations corresponding to clinically administered dosages and associated local tissue concentrations in the presence and absence of physiological mechanical compressive strain, occurring during orthodontic tooth movement. We found a significant inhibition of prostaglandin E_2_ synthesis at both concentrations tested according to the known pharmacodynamic mechanism of etoricoxib, whereas expression of other inflammatory mediators in hPDL fibroblasts such as COX-2 and IL-6 as well as RANK-L-/OPG-expression and hPDL-mediated osteoclastogenesis and angiogenesis (VEGF-A) was not significantly affected. By contrast, etoricoxib, particularly at the high concentration, had a stimulating effect on collagen synthesis and remodeling of the extracellular matrix of the periodontal ligament. Furthermore, increased anabolic activity regarding bone formation is indicated by the observed elevated expression of alkaline phosphatase (ALPL), which is an important biomarker for bone formation and generally reported to have an increased biological activity in the periodontal ligament compared to other connective tissues [42]. With the exception of a slight significant increase in necrotic cells during force application at the high etoricoxib concentration tested, no adverse effects on hPDL fibroblasts regarding apoptosis, necrosis, cell viability, or cytotoxicity were detected, confirming previous reports of its favorable side effect profile [27].

For orthodontic tooth movement, mechanical forces are applied to teeth, creating pressure and tension zones within the periodontal ligament. Via the extracellular matrix and most likely integrin (focal adhesions) as well as cAMP and IP3 signaling [11], hPDL fibroblasts put under mechanical strain react by changing their expression pattern activating various signaling pathways such as MAP kinase ERK, p38, JNK, and NF-*κ*B signaling [43, 44], leading to increased synthesis of cyclooxygenase 2 (COX-2) [45] and thus prostaglandins from arachidonic acid within 15 minutes after force application [32], which was confirmed by our results. Hong et al. [46] could show that the mechanical deformation of the cellular membrane itself leads to increased prostaglandin synthesis, most likely due to an exposure of phospholipids, enabling increased accessibility for MAPK-phosphorylated and activated phospholipase A2 to synthesize arachidonic acid [6], thus further promoting prostaglandin, prostacyclin, and thromboxane production by cyclooxygenases 1 and 2 as well as leukotrienes by lipoxygenase 5 [47]. Prostaglandins [30, 31] as well as leukotrienes [48, 49] have been shown to be important autocrine and paracrine mediators of mechanical (orthodontic) strain, leading to increased synthesis of proinflammatory cytokines such as IL-1, IL-6—as shown in our study—and chemokines such as IL-8 [50–52], attracting lymphocytes from peripheral blood [53], which are the primary sources of RANK-L in periodontitis [54]. This immigration of immune cells, which further propagates inflammation and osteoclastogenesis, is promoted by an increased expression of vascular endothelial growth factor (VEGF-A) by mechanically stressed hPDL fibroblasts, as evidenced by our results, furthering angiogenesis and vasodilatation [55]. In addition, prostaglandins and leukotrienes stimulate osteoclastogenesis and bone resorption [31, 49] as well as induce the expression of soluble and membrane-associated RANK-L, which has been shown to be PG-E_2_ dependent [30], with expression of its soluble decoy receptor osteoprotegerin (OPG) being downregulated [56], confirmed by our own data. The interaction of the RANK ligand with the RANK receptor as a major stimulating factor for osteoclast differentiation and activity [7] then enables bone resorption and orthodontic movement of teeth through the alveolar bone, as evidenced by the significantly increased osteoclastogenesis observed in hPDL RAW267.4-coculture [38]. On the other hand, several studies have also shown that mechanical strain on hPDL fibroblasts also increases the expression of matrix metalloproteinases (MMP), their inhibitors (TIMP) [57], and collagen synthesis [58], thus enabling increased remodeling of the extracellular matrix [6, 11] promoting cell proliferation as well as osteoblastogenesis and bone formation, which we could corroborate in our study.

The observed significant inhibition of prostaglandin E_2_ synthesis by etoricoxib at both concentrations evaluated is in accordance with its known pharmacodynamic mechanism, which selectively inhibits inflammation-induced cyclooxygenase 2 (COX-2) with a preference of 344 : 1 compared to the constitutively expressed COX isoform 1 [24, 25, 27, 35]. Both cyclooxygenases catalyze the synthesis of prostaglandins from arachidonic acid [47]. Despite prostaglandins being potent stimulators of proinflammatory cytokine expression [50–52] as well as osteoclastogenesis [31], their significant inhibition by both etoricoxib concentrations tested did not significantly affect COX-2 and IL-6 gene expression nor the RANKL-OPG ratio or osteoclastogenesis in coculture of hPDL fibroblasts with RAW264.7 cells. A possible explanation for this phenomenon could be the extremely high COX-2 selectivity of etoricoxib, which basically only targets COX-2 leaving COX-1 activity unaffected, as well as the presence of other arachidonic acid-metabolizing enzymes such as lipoxygenase 5 as well as proinflammatory cytokines. The increasingly available arachidonic acid from membrane lipid exposure [46] and MAPK-initiated phospholipase A2 expression [6] during mechanical strain could thus be metabolized to other prostaglandins than PG-E_2_ by COX-1 as well as by lipoxygenase 5 to leukotrienes, which have been shown to be potent stimulators of osteoclastogenesis as well [49], compensating for the loss of COX-2 activity and prostaglandin E_2_ synthesis. Furthermore, the observed etoricoxib-unaffected force-elevated expression of IL-6 or other cytokines and chemokines such as IL-8 might compensate for the loss of prostaglandin activity, since IL-6 and IL-11 have been shown to directly stimulate osteoclast activity by a RANKL-independent mechanism [59] and elevated chemokine expression might attract RANKL-producing T-lymphocytes into the periodontal ligament [53, 54].

COX-2 selectivity of NSAID and its effects on osteoclastogenesis and orthodontic tooth movement is still controversial in the current literature [15, 19, 20, 23]. This is most likely due to various clinically more or less relevant dosages investigated with pharmacodynamic effects being dose dependent as well as substantially differing COX-2 selectivity of the various coxibs available with celecoxib and parecoxib being only 30 or 60 times more selective, respectively. A previous study on the partially COX-2-selective (11 : 1) NSAID meloxicam [10] showed a less pronounced inhibitory effect on hPDL fibroblast-mediated osteoclastogenesis than conventional nonselective NSAID, but a more profound effect than most coxibs tested up to the highly selective etoricoxib of this study having no effects. Based on the available evidence and the results of the present study, it thus seems likely that increasing COX-2 selectivity is associated with less effects on hPDL fibroblast-mediated osteoclastogenesis and thus orthodontic tooth movement. Further studies directly comparing NSAID of various selectivity in comparable, clinically relevant dosages and concentrations should therefore be performed to clarify and corroborate this hypothesis.

No previous reports on the effects of etoricoxib on hPDL or other fibroblasts have been found in the literature. Available animal studies in a rat model of periodontitis, however, have reported suppressive effects of etoricoxib on inflammation, osteoclastogenesis, and alveolar bone loss [60, 61], which could, however, not be replicated in man [62], corresponding to our own results on human PDL fibroblasts. In contrary to the less COX-2-selective celecoxib [63], etoricoxib had no negative impact on alveolar bone healing in rats [64], whereas a reported antiangiogenetic effect in experimental lung cancer [65] was not observed at the etoricoxib concentrations used in our study.

Available literature on the effects of other coxibs on hPDL or other fibroblasts is scarce with only a few reports available. Römer et al. showed a distinct inhibitory effect of the distinctly less COX-2-selective celecoxib on the expression of inflammatory cytokines and RANK-L by hPDL fibroblasts stimulated with pressure and bacterial toxins [41]. Also, in synovial fibroblasts from the temporomandibular joint of patients with rheumatoid arthritis, celecoxib reduced COX-2 and IL-6 expression by suppression of PG-E_2_ synthesis during stimulation of cells with PG-E_2_/IL-1*β* [66]. In contrary to our own observations, an inhibition of cell proliferation and collagen synthesis of celecoxib-stimulated NIH/3T3 fibroblasts via ERK1/2 and SMAD2/3 phosphorylation was reported [67] as well as a proapoptotic effect on synovial fibroblasts [68]. Further investigations have however corroborated that the reported antiproliferative and proapoptotic effects of celecoxib are not class effects of coxibs [68, 69], which might explain the differing results to etoricoxib tested in this study limiting comparability.

For our study, we used an established and valid in vitro model for the simulation of physiological orthodontic compressive forces. 2 g/cm^2^ have been shown to elicit the maximum cellular response of hPDL fibroblasts under compressive strain with 4 g/cm^2^ causing cellular damage [30]. To support sufficient nutrition of cells beneath the sterilized glass plate, which is ensured by diffusion from the lateral sides, we placed it into full medium for 15 min prior to the start of experiments to ensure surface saturation with proteins [41].

In coculture experiments on hPDL-mediated osteoclastogenesis, an established coculture model (RAW264.7 macrophages) was used [70–72]; however, both mononucleated and multinucleated TRAP-positive cells, corresponding to osteoclast precursor and osteoclast-like cells [39], were considered for analysis, which needs to be taken into account when comparing our results to those of other studies.

We primarily used LDH assays to assess cytotoxicity based on the cell membrane damage evaluated in this test, whereas MTT assays were used to assess cell viability, that is, metabolic activity, which does not necessarily correspond to cell death but reduced cell function and metabolism. Since LDH assays did not show a significant influence of either compressive force or etoricoxib regarding cytotoxicity, this observation concurs well with our FACS results. MTT assays also did not yield any significant effect of etoricoxib on cell viability, whereas the compressive force applied significantly reduced cell viability, that is, mitochondrial enzymatic activity, as assessed by the MTT assay. We thus hypothesize that either the mechanical stress itself [73] or an accompanying lack of oxygen [74] in our in vitro model, which is also supposed to occur during orthodontic tooth movement in vivo [75], most likely attenuated PDL fibroblast cell metabolism to a significant degree, whereas cell death was not significantly induced.

Although a direct clinical extrapolation of biological significance to the clinical situation is not possible, we attempted to maximize generalizability and translatability of our results by choosing etoricoxib concentrations, which are likely to occur in the clinical context locally within the periodontal ligament microenvironment during medication. These were supposed to correspond to steady-state blood plasma concentrations of normal (90 mg/day) and subtoxic (150 mg/day) etoricoxib dosing reached in man [36], since the extracellular fluid within the periodontal ligament is a plasma transudate from the periodontal capillaries [76]. Thus, the assumption made that local etoricoxib concentrations in the PDL microenvironment correspond to etoricoxib concentrations reached in blood plasma should be valid. Approximated mean steady-state plasma concentrations of etoricoxib, which shows linear pharmacokinetics in man in dosages from 30-240 mg/day [35], were calculated from known 24 h AUC values for multiple dosing of 120 mg/day (37.83 *μ*g·h/ml) by dividing the extrapolated AUC_24h_ for normal 90 mg/day (28.37 *μ*g·h/ml) and subtoxic 150 mg/day (47.29 *μ*g·h/ml) dosing by 24 h [35], resulting in mean blood plasma concentrations of 1.18 *μ*g/ml (3.29 *μ*M, molar mass etoricoxib = 358.84 g/mol) and 1.97 *μ*g/ml (5.49 *μ*M), respectively, which were used as experimental etoricoxib concentrations in the present study.

## 5. Conclusions

Based on our results, it seems likely that etoricoxib medication, that is a highly selective inhibition of COX-2, at cell medium concentrations corresponding to clinically administered dosages and associated local tissue concentrations within the periodontal ligament does not have substantial effects on hPDL fibroblast-mediated periodontal inflammation, extracellular matrix remodeling, RANK-L/OPG expression, and osteoclastogenesis during simulated orthodontic compressive force application. Due to its also favorable side effect profile and high analgesic efficacy, particularly for orthodontic pain, it could therefore be a valid analgesic during orthodontic treatment.

## Figures and Tables

**Table 1:** RT-qPCR primer data for target genes as well as reference genes (PPIB, RPL22) used for normalization of gene expression. Primers were not modified and synthesized and purified by Eurofins MWG Operon LLC (Huntsville, AL, USA; High Purity Salt Free Purification HPSF®).

Gene symbol	Accession number (NCBI GenBank)	Sequence 5′-forward primer-3′	Sequence 5′-reverse primer-3′
PPIB	NM_000942.4	TTCCATCGTGTAATCAAGGACTTC	GCTCACCGTAGATGCTCTTTC
RPL22	NM_000983.3	TGATTGCACCCACCCTGTAG	GGTTCCCAGCTTTTCCGTTC
COX-2	NM_000963.3	GAGCAGGCAGATGAAATACCAGTC	TGTCACCATAGAGTGCTTCCAAC
IL6	NM_000600.3	TGGCAGAAAACAACCTGAACC	CCTCAAACTCCAAAAGACCAGTG
ALPL	NM_000478.4	ACAAGCACTCCCACTTCATCTG	GGTCCGTCACGTTGTTCCTG
VEGFA	NM_001171623.1	TGCAGACCAAAGAAAGATAGAGC	ACGCTCCAGGACTTATACCG
P4HA1	NM_000917.3	GCTCTCTGGCTATGAAAATCCTG	GTGCAAAGTCAAAATGGGGTTC
COL1A2	NM_000089.3	AGAAACACGTCTGGCTAGGAG	GCATGAAGGCAAGTTGGGTAG
FN1	NM_212482.1	GCCAGTCCTACAACCAGTATTCTC	GCTTGTTCCTCTGGATTGGAAAG

## Data Availability

All data are publically available either as supplementary information to this article or upon request.

## Supplementary Materials

Supplementary Materials Supplementary Figure 1: characterization of isolated hPDL fibroblasts. Spindle-shaped cell morphology and expression of hPDL fibroblast-specific marker genes. Abbreviations: see Supplementary Table 1. Supplementary Table 1: gene, primer, and amplicon data used for PCR amplification in hPDL fibroblast characterization. Supplementary Table 2: detailed information on RT-qPCR gene, primer, target, and amplicon data for target genes as well as reference genes (PPIB, RPL22) used for normalization
