# Supplementary material for: Effects of the Highly COX-2-Selective Analgesic NSAID Etoricoxib on Human Periodontal Ligament Fibroblasts during Compressive Orthodontic Mechanical Strain
Source: Mediators Inflamm. 2019 Mar 10;2019:2514956. doi: 10.1155/2019/2514956 (PMC6431464; doi:10.1155/2019/2514956)
Supplement: Supplementary Materials — Supplementary Figure 1: characterization of isolated hPDL fibroblasts. Spindle-shaped cell morphology and expression of hPDL fibroblast-specific marker genes. Abbreviations: see Supplementary Table 1. Supplementary Table 1: gene, primer, and amplicon data used for PCR amplification in hPDL fibroblast characterization. Supplementary Table 2: detailed information on RT-qPCR gene, primer, target, and amplicon data for target genes as well as reference genes (PPIB, RPL22) used for normalization [file 2514956.f1.pdf]

Supplementary Materials

Supplemental information on RT-qPCR methodology

RNA isolation and quality assessment as well as RT-qPCR were performed as described before according to MIQE guidelines [34]. Total RNA was retrieved from hPDL fibroblasts by administering 1 ml peqGOLD TriFastTM (PEQLAB Biotechnology GmbH, Erlangen, Germany) per well and further handling according to the manufacturer's instructions. The obtained RNA pellet was eluted in 25 µl nuclease-free water (T143, Bioscience-Grade, Carl Roth GmbH & Co. KG, Karlsruhe, Germany) and immediately cooled on ice. As shown before [34], this protocol ensured excellent RNA integrity (RIN, 28S/18S ratio) as well as genomic-DNA-free samples. Purity and amount of the eluted total RNA was determined by its optical density (OD) at 280 nm, 260 nm and 230 nm (NanoDrop ND-2000, Thermo Fisher Scientific, Schwerte, Germany) with 1 OD_{260nm} corresponding to 40 ng/µl total RNA. An $OD_{260nm/280nm}$ ratio of >1.8 confirmed protein-free RNA and an $OD_{260nm/230nm}$ ratio of >2.0 phenol-/ethanol-free RNA [34].

A standardized quantity of 1 µg RNA per sample was transcribed in cDNA employing 0.1 nmol of an oligo-dT18 primer (1 µl, SO131, Thermo Fisher Scientific, Schwerte, Germany), 0.1 nmol of a random hexamer primer (1 µl, SO142, Thermo Fisher Scientific), 40 nmol dNTP mix (1 µl, 10 nmol/dNTP, Roti®-Mix PCR3, L785.2, Carl Roth GmbH & Co. KG) and 4 µl 5× M-MLV-buffer (M1705, Promega, Fitchburg, WI, USA) ad 20 µl nuclease-free H_2O (T143,Carl Roth GmbH & Co. KG). We incubated this reaction mix for 3 min at 70°C with immediate cooling on ice for RNA denaturation. After adding 200 U (1 µl) reverse transcriptase (M1705, Promega) and 40 U (1 µl) of an RNase inhibitor (EO0381, Thermo Fisher Scientific), we continued incubation for 60 min at 37°C and heat-inactivated the reverse transcriptase (95°C, 2 min) with cDNA stored until use at −20°C. To minimize experimental variation, all samples were processed into cDNA at the same time.

A Mastercycler® ep realplex-S thermocycler (Eppendorf AG, Hamburg, Germany) was used for RT-qPCR as well as 96-well PCR plates (TW-MT, 712282, Biozym Scientific GmbH, Hessisch Oldendorf, Germany) in combination with BZO Seal Filmcover sheeting (712350, Biozym Scientific GmbH) as described before [34]. Each reaction mix comprised 7.5µl SYBR®Green JumpStart TM Taq ReadyMix TM (S4438; Sigma–Aldrich, Munich, Germany) as well as 1.5 µl of the respective cDNA-solution (dilution 1:10) and 7.5 pmol (0.75 µl) of the respective primer pair (3.75 pmol/primer). To achieve a total amount of 15 µl,

nuclease-free H_2O (T143, Carl Roth GmbH & Co. KG) was added accordingly with all components (except cDNA) prepared as a master-mix to minimize manual-pipetting-related technical errors. cDNA amplification was performed in 45 cycles (initial heat activation 95°C/5 min, per cycle 95°C/10 s denaturation, 60°C/8 s annealing, 72°C/8 s extension). At the end of each extension step SYBR Green I fluorescence was quantified at 521 nm and C_q values identified as second derivative maximum of the fluorescence signal curve employing the software realplex (version 2.2, Eppendorf AG, CalqPlex algorithm, Automatic Baseline, Drift Correction On). Normalization of target genes for assessment of relative gene expression was based on two reference genes (RPL22/ PPIB), which were validated before for hPDL fibroblasts and the *in vitro* model used [34]. We calculated relative gene expression as $2^{-\Delta C_q}$ [37] with $\Delta C_q = C_q$ (target gene) – C_q (mean RPL22/PPIB).

RT-qPCR primer design was performed according to MIQE quality criteria as described before [34] as intron-flanking and gene-specific (Table 1), utilizing NCBI PrimerBLAST and other online freeware programs, also ensuring sufficient absence of secondary structures and dimers at annealing temperature. Primers were not modified and synthesized and purified by Eurofins MWG Operon LLC (Huntsville, AL,USA; High Purity Salt Free Purification HPSF®). A no-template-control (NTC) without cDNA was amplified for each primer pair and qPCR run to ensure absence of primer dimers or contaminating DNA. Validation of RT-qPCR specifity was conducted as previously reported by melting curve analysis and agarose gel electrophoresis [34].

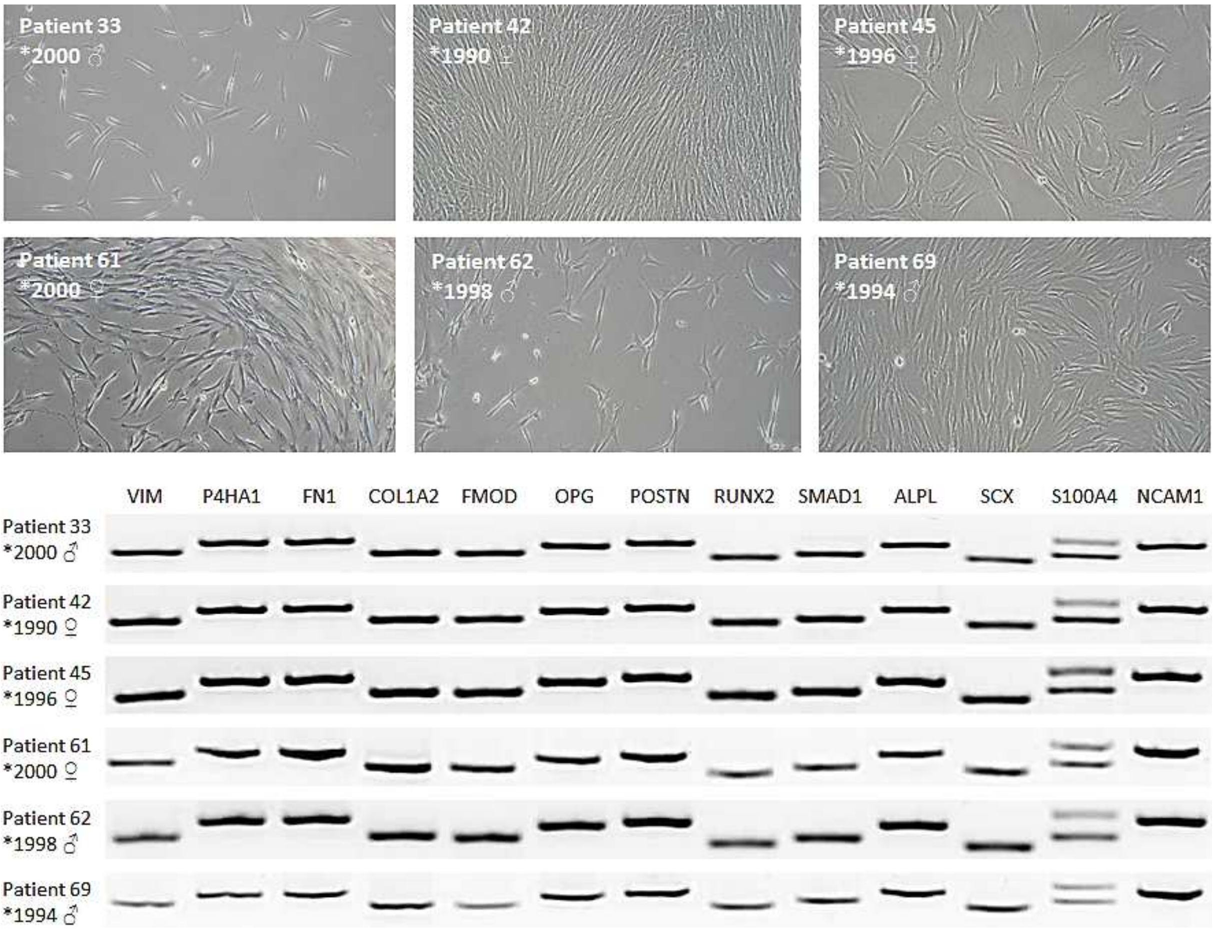

Supplementary Figure 1. Characterization of isolated hPDL fibroblasts. Spindle-shaped cell morphology and expression of hPDL-fibroblast-specific marker genes. Abbreviations see Supplementary Table 1.

Supplementary Table 1. Gene, primer and amplicon data used for PCR-amplification in hPDL-fibroblast characterization.

**Gene symbol**	**Gene name** (Homo sapiens)	**Accession Number** (NCBI GenBank)	**Chromosomal location** (length)	**5´-forward primer-3´** (length / T_m / %GC / max. ΔG Hairpin &Self-Dimer / Self-Comp. / Self-3'-Comp.)	**5´-reverse primer-3´** (length / T_m / %GC / max. ΔG Hairpin &Self-Dimer / Self-Comp. / Self-3'-Comp.)	**Primer Location** (max. ΔG Cross-Dimer)	**Amplicon** (length, %GC, T_m, SSAT)	**Amplicon location** (bp of Start/Stop)	**Intron-spanning** (length)	**In silico qPCR specifity**	**Variants targeted (Transcript /Splice)**
**VIM**	vimentin	NM_003380.3	10p13 (2151bp)	CTGGATTCACTCCCTCTGGTTG (22bp / 62.9°C / 54.6% / -1.3 / 5 / 0)	CGTGATGCTGAGAAGTTTCGTTG (23bp / 63.1°C / 47.8% / -0.6 / 4 / 0)	exon 8/9 (-2.6)	106bp, 44.3%, 82.3%, no SSAT	1695/1800	Yes (850bp)	Yes (BLAST/ UCSC)	Yes
**P4HA1**	prolyl 4-hydroxylase, alpha polypeptide I	NM_000917.3	10q22.1 (2860bp)	GCTCTCTGGCTATGAAAATCCTG (23bp / 61.7°C / 47.8% / 0.0 / 2 / 2)	GTGCAAAGTCAAAATGGGGTTC (22bp / 61.3°C / 45.5% / -3.4 / 4 / 0)	exon 13/14 (-0.9)	146bp, 41.1%, 82.2°C, no SSAT	1396/1541	Yes (13371bp)	Yes (BLAST/ UCSC)	Yes
**FN1**	fibronectin 1	NM_212482.1	2q34 (8815bp)	GCCAGTCCTACAACCAGTATTCTC (24bp / 63.2°C / 50.0% / -0.3 / 4 / 2)	GCTTGTTCCTCTGGATTGGAAAG (23bp / 62.3°C / 47.8% / -2.5 / 4 / 1)	exon 45/46 (-3.0)	150bp, 42.7%, 83.1°C, no SSAT	7579/7728	Yes (342bp)	Yes (BLAST/ UCSC)	Yes
**COL1A2**	collagen, type I, alpha 2	NM_000089.3	7q22.1 (5411bp)	AGAAACACGTCTGGCTAGGAG (21bp / 61.9°C / 52.4% / -3.3 / 4 / 2)	GCATGAAGGCAAGTTGGGTAG (21bp / 62.0°C / 52.4% / -2.3 / 5 / 0)	exon 50/51 (-0.7)	105bp, 44.8%, 83.3°C no SSAT	4139/4243	Yes (710bp)	Yes (BLAST/ UCSC)	Yes
**FMOD**	fibromodulin	NM_002023.4	1q32 (3271bp)	AGTCAACACCAACCTGGAGAAC (22bp / 62.9°C / 50.0% / -1.5 / 3 / 0)	GAAGTTCACGACGTCCACCAC (21bp / 63.9°C / 57.1% / -6.5 / 6 / 3)	exon 2/3 (-2.8)	97bp, 51.6%, 85.7°C no SSAT	1334/1430	Yes (4797bp)	Yes (BLAST/ UCSC)	Yes
**TNFRSF11B (OPG)**	tumor necrosis factor receptor superfamily, member 11b (osteoprotegerin)	NM_002546.3	8q24 (2354bp)	TGTCTTTGGTCTCCTGCTAACTC (23bp / 62.5°C / 47.8% / 0.0 / 2 / 0)	CCTGAAGAATGCCTCCTCACAC (22bp / 63.4°C / 54.6% / -0.9 / 4 / 0)	exon 3/4 (-1.8)	124bp, 42.7%, 83.1°C no SSAT	824/947	Yes (2019bp)	Yes (BLAST/ UCSC)	Yes
**POSTN**	periostin	NM_006475.2	13q13.3 (3390bp)	AGACACACCCGTGAGGAAG (19bp / 61.4°C / 57.9% / -1.3 / 4 / 0)	GGTCAGGTTATTGACTTAGGGTTG (24bp / 61.6°C / 45.8% / -2.6 / 4 / 0)	exon 23/24 (-3.4)	136bp, 39.4%, 81.9°C no SSAT	2548/2683	Yes (1148bp)	Yes (BLAST/ UCSC)	Yes
**RUNX2**	runt related transcription factor 2	NM_001024630.3	6p21 (5553bp)	CAGTAGATGGACCTCGGGAAC (21bp / 62.1°C / 57.1% / 0.0 / 3 / 0)	TGAGGCGGTCAGAGAACAAAC (21bp / 63.0°C / 52.4% / -0.9 / 3 / 0)	exon 5/6 (-3.1)	81bp, 50.6%, 83.7°C no SSAT	869/949	Yes (53889bp)	Yes (BLAST/ UCSC)	Yes
**SMAD1**	SMAD family member 1	NM_005900.2	4q31 (3056bp)	AGCAGCACCTACCCTCACTC (20bp / 63.8°C / 60.0% / 0.0 / 3 / 0)	CTTCAGGAGGCAGGTAAGCAG (21bp / 62.9°C / 57.1% / -0.5 / 3 / 1)	exon 4/5 (-2.9)	97bp, 60.8%, 90.4°C, no SSAT	1014/1110	Yes (2520bp)	Yes (BLAST/ UCSC)	Yes
**ALPL**	alkaline phosphatase, liver/bone/kidney	NM_000478.4	1p36.12 (2606bp)	ACAAGCACTCCCACTTCATCTG (22bp / 63.1°C / 50.0% / -0.5 / 3 / 2)	GGTCCGTCACGTTGTTCCTG (20bp / 63.6°C / 60.0% / -3.3 / 5 / 1)	exon 7-8/9 (-2.1)	132bp, 56.1%, 89.5°C, no SSAT	1045/1176	Yes (3290bp)	Yes (BLAST/ UCSC)	Yes
**SCX**	scleraxis bHLH transcription factor	NM_001080514.2	8q24.3 (1027bp)	CCAGCCCAAACAGATCTGCAC (21bp / 64.3°C / 57.1% / -7.9 / 8 / 2)	TGCGAATCGCTGTCTTTCTGTC (22bp / 63.9°C / 50.0% / -4.2 / 7 / 1)	exon 1/2 (-3.8)	83bp, 54.2%, 86.6°C, no SSAT	575/657	Yes (923bp)	Yes (BLAST/ UCSC)	Yes
**S100A4**	S100 calcium binding protein A4	NM_002961.2	1q21 (512bp)	TCTCTACAACCCTCTCTCCTCAG (23bp / 62.6°C / 52.2% / 0.0 / 3 / 3)	GGAAGGTGGACACCATCACATC (22bp / 63.4°C / 54.6% / -3.2 / 8 / 1)	exon 1/3 (-1.5)	108bp, 54.1%, 87.8°C no SSAT	11/118	Yes (943bp)	Yes (BLAST/ UCSC)	Yes
**NCAM1**	neural cell adhesion molecule 1 (NCAM1)	NM_000615.6	11q23.1 (5977bp)	CTCCCACCAACCATCATCTGG (21bp / 62.9°C / 57.1% / -1.5 / 3 / 2)	CAGGATTCTGCCCTCACAGC (20bp / 63.2°C / 60.0% / -1.3 / 6 / 2)	exon 4/5 (-1.3)	150bp, 49.3%, 86.6°C no SSAT	799/948	Yes (376bp)	Yes (BLAST/ UCSC)	Yes

T_m = melting temperature of primer/specific PCR product (amplicon); %GC = guanine/cytosine content; bp = base pairs; Comp. = Complementarity; SSAT = secondary structure at annealing temperature

Supplementary Table 2. Detailed information on RT-qPCR gene, primer, target and amplicon data for target genes as well as reference genes (PPIB, RPL22) used for normalization of gene expression.

**Gene symbol**	**Gene name** (Homo sapiens)	**Accession Number** (NCBI GenBank)	**Chromosomal location** (length)	**5´-forward primer-3´** (length / T_m / %GC / max. ΔG Hairpin &Self-Dimer / Self-Comp. / Self-3'-Comp.)	**5´-reverse primer-3´** (length / T_m / %GC / max. ΔG Hairpin &Self-Dimer / Self-Comp. / Self-3'-Comp.)	**Primer Location** (max. ΔG Cross-Dimer)	**Amplicon** (length, %GC, T_m, SSAT)	**Amplicon location** (bp of Start/Stop)	**Intron-spanning** (length)	**In silico qPCR specifity**	**Variants targeted (Transcript /Splice)**
**PPIB**	peptidylprolyl isomerase A	NM_000942.4	15q21-q22 (1045bp)	TTCCATCGTGTAATCAAGGACTTC (24bp / 61.3°C / 41.7% / -1.3 / 4 / 2)	GCTCACCGTAGATGCTCTTTC (21bp / 61.2°C / 52.4% / -0.7 / 4 / 0)	exon 3/4 (-2.1)	88bp, 53.4%, 86.1°C, no SSAT	446/533	Yes (3194bp)	Yes (BLAST /UCSC)	Yes
**RPL22**	ribosomal protein L22	NM_000983.3	1p36.31 (2099bp)	TGATTGCACCCACCCTGTAG (20bp / 62.2°C / 55.0% / -3.4 / 4 / 2)	GGTTCCCAGCTTTTCCGTTC (20bp / 61.8°C / 55.0% / -3.0 / 4 / 0)	exon 2/3 (-1.5)	98bp, 44.9%, 83.8°C, no SSAT	115/212	Yes (4597bp)	Yes (BLAST /UCSC)	Yes
**COX2**	prostaglandin-endoperoxide synthase 2	NM_000963.3	1q25.2-25.3 (4507bp)	GAGCAGGCAGATGAAATACCAGTC (24bp / 62.7°C / 50.0% / 0.0 / 2 / 2)	TGTCACCATAGAGTGCTTCCAAC (23bp / 60.6°C / 47.8% / -1.3 / 4 / 0)	exon 8/9 (-3.2)	131bp, 42.0%, 82.9°C, no SSAT	1457/1587	Yes (486bp)	Yes (BLAST /UCSC)	Yes
**IL6**	interleukin 6	NM_000600.3	7p21 (1201bp)	TGGCAGAAAACAACCTGAACC (21bp / 57.9°C / 47.6% / -1.1 / 3 / 0)	CCTCAAACTCCAAAAGACCAGTG (23bp / 60.6°C / 47.8% / -0.8 / 3 / 3)	exon 2/3 (-1.5)	117bp, 43.6%, 83.7°C, no SSAT	370/486	Yes (704bp)	Yes (BLAST /UCSC)	Yes
**ALPL**	alkaline phosphatase, liver/bone/kidney	NM_000478.4	1p36.12 (2606bp)	ACAAGCACTCCCACTTCATCTG (22bp / 60.3°C / 50.0% / -0.5 / 3 / 2)	GGTCCGTCACGTTGTTCCTG (20bp / 61.4°C / 60.0% / -3.3 / 5 / 1)	exon 7-8/9 (-2.1)	132bp, 56.1%, 89.5°C, no SSAT	1045/1176	Yes (3290bp)	Yes (BLAST /UCSC)	Yes
**VEGFA**	vascular endothelial growth factor A	NM_001171623.1	6p12 (3677bp)	TGCAGACCAAAGAAAGATAGAGC (23bp / 58.9°C / 43.5% / -3.4 / 4 / 2)	ACGCTCCAGGACTTATACCG (20bp / 59.4°C / 55.0% / -1.3 / 5 / 2)	exon 5-6/7 (-3.3)	107bp, 43.9%, 83.7°C, no SSAT	1426/1532	No	Yes (BLAST /UCSC)	Yes
**P4HA1**	prolyl 4-hydroxylase, alpha polypeptide I	NM_000917.3	10q22.1 (2860bp)	GCTCTCTGGCTATGAAAATCCTG (23bp / 61.7°C / 47.8% / 0.0 / 2 / 2)	GTGCAAAGTCAAAATGGGGTTC (22bp / 61.3°C / 45.5% / -3.4 / 4 / 0)	exon 13/14 (-0.9)	146bp, 41.1%, 82.2°C, no SSAT	1396/1541	Yes (13371bp)	Yes (BLAST /UCSC)	Yes
**COL1A2**	collagen, type I, alpha 2	NM_000089.3	7q22.1 (5411bp)	AGAAACACGTCTGGCTAGGAG (21bp / 59.8°C / 52.4% / -3.3 / 4 / 2)	GCATGAAGGCAAGTTGGGTAG (21bp / 59.8°C / 52.4% / -2.3 / 5 / 0)	exon 50/51 (-0.7)	105bp, 44.8%, 83.3°C no SSAT	4139/4243	Yes (710bp)	Yes (BLAST /UCSC)	Yes
**FN1**	fibronectin 1	NM_212482.1	2q34 (8815bp)	GCCAGTCCTACAACCAGTATTCTC (24bp / 63.2°C / 50.0% / -0.3 / 4 / 2)	GCTTGTTCCTCTGGATTGGAAAG (23bp / 62.3°C / 47.8% / -2.5 / 4 / 1)	exon 45/46 (-3.0)	150bp, 42.7%, 83.1°C, no SSAT	7579/7728	Yes (342bp)	Yes (BLAST /UCSC)	Yes

T_m = melting temperature of primer/specific qPCR product (amplicon); %GC = guanine/cytosine content; bp = base pairs; Comp. = Complementarity; SSAT = secondary structure at annealing temperature
